# Comparative effectiveness of conservative and surgical interventions for toe walking in children with Autism Spectrum Disorder: a systematic review

**DOI:** 10.3389/fmed.2026.1832930

**Published:** 2026-07-20

**Authors:** Emanuele Perricone, Alessia Caldaci, Giovanni Cacciaguerra, Alessandra di Nora, Claudia Parano, Vito Pavone

**Affiliations:** 1 Department of General Surgery and Medical Surgical Specialties, Section of Orthopedics and Traumatology, A.O.U.P. Policlinico Rodolico-San Marco, University of Catania, Catania, Italy; 2 Institute for Biomedical Research and Innovation (IRIB), Italian National Research Council (CNR), Catania, Italy; 3 Units of Pediatrics, Lentini Hospital, Siracusa, Italy; 4 Postgraduate Training Program in Plastic, Reconstructive and Aesthetic Surgery, Department of General Surgery and Medical-Surgical Specialties, University of Palermo, Palermo, Italy

**Keywords:** Autism Spectrum Disorders, conservative treatment, outcome hierarchy, rehabilitation, surgical treatment, toe walking

## Abstract

**Introduction:**

Toe walking (TW) is frequently observed in children with Autism Spectrum Disorder (ASD) and may be associated with sensory and motor dysfunctions affecting mobility and quality of life. This systematic review aimed to synthesize current evidence on reported outcomes of conservative and surgical interventions, with attention to unimodal and multimodal conservative strategies.

**Methods:**

A PRISMA-based systematic review was conducted using PubMed, Scopus, and Google Scholar. Studies reporting interventions for toe walking in children with ASD were included. Study characteristics, treatment approaches, outcome definitions, and reported improvement rates were extracted. Because of substantial heterogeneity, findings were summarized descriptively according to a predefined outcome hierarchy.

**Results:**

Eight studies including 424 ASD patients, aged 2–16 years, were included. Conservative and multimodal conservative treatment arms generally reported numerically higher improvement values than surgical and unimodal approaches, respectively. However, these summaries were unweighted, study-level descriptions based on heterogeneous and non-equivalent outcome measures.

**Discussion:**

Current evidence is of very low certainty and does not allow conclusions regarding comparative effectiveness or treatment superiority. Findings should be considered descriptive and hypothesis-generating only. Further high-quality prospective studies using standardized clinical outcomes and long-term follow-up are required.

## Introduction

Autism Spectrum Disorder (ASD) encompasses a group of neurodevelopmental conditions characterized by persistent deficits in social communication and interaction, alongside restricted and repetitive patterns of behavior, interests, or activities, as defined by the Diagnostic and Statistical Manual of Mental Disorders, Fifth Edition (DSM-5) (1). The core characteristics of ASD are impairments in communication, social interaction, and restricted repetitive and stereotyped behaviors. Moreover, children with ASD often display co-morbid disorders, such as intellectual disability and language impairment (2). Beyond these core features, individuals with ASD frequently present with sensory processing abnormalities and motor dysfunctions, which may significantly influence daily activities and overall quality of life (3, 4). Although the motor profile in individuals with ASD is heterogeneous and not all individuals will exhibit motor difficulties (5), a growing body of evidence indicates that motor impairments are commonly present in ASD. These impairments may include delays in achieving motor milestones (6, 7), difficulties with coordination (8), abnormalities in reach-to-grasp movements (3), deficits in both gross and fine motor skills (9), difficulty in planning and imitating movements (10) and impaired postural control (11). Among the various motor anomalies reported in children with ASD, toe walking, defined as a persistent forefoot gait without initial heel strike, has been consistently observed with higher prevalence compared to neurotypical peers. Reports suggest that up to 41%−68% of children with ASD may exhibit toe walking or similar gait abnormalities (12, 13). Although toe walking can be idiopathic in nature, its occurrence in ASD is often multifactorial, possibly arising from deficits in sensory integration, proprioceptive dysfunction, altered motor planning, or retained primitive reflexes such as the tonic labyrinthine reflex (14, 15). The clinical significance of toe walking in ASD extends beyond biomechanics. Persistent equinus gait can lead to musculoskeletal complications including Achilles tendon shortening, joint instability, and postural deviations. An equinus gait lasting longer than 3 months after onset of independent ambulation in the absence of other neuroorthopedic disorders has been characterized as persistent toe walking and associated with developmental delay and language disorders (15). Furthermore, it may reflect or exacerbate sensory sensitivities and behavioral rigidity commonly observed in ASD, potentially limiting social participation and functional mobility (13). A wide array of therapeutic strategies has been proposed to address toe walking in children with and without ASD. Conservative treatments include physical therapy (16), coordination and balance exercises (17), orthotic use, casting (18), botulinum toxin injections (19), and ankle-foot orthoses (AFOs) (20), sensorimotor (21) or aquatic therapy (22). Multimodal rehabilitation programs, combining motor training and behavioral strategies, have shown promising results (13, 23). In refractory cases or when fixed contractures are present, surgical interventions such as Achilles tendon lengthening may be considered (24, 25). Tailored rehabilitation plans are essential to address individual sensory and motor profiles in ASD. While single-component approaches may yield partial benefit, emerging evidence suggests a potential role for multimodal conservative programs tailored to the sensory-motor profiles of autistic individuals (16, 21). This systematic review aims to synthesize the current evidence regarding treatment modalities for toe walking in ASD, providing a descriptive comparison of conservative and surgical interventions and evaluating the reported outcomes of unimodal and multimodal conservative strategies.

## Material and methods

### Search selection

A systematic review was conducted in accordance with the Preferred Reporting Items for Systematic Reviews and Meta-Analyses (PRISMA) guidelines (26) by independent researchers (EP, AC, GC, Ad, CP), under the supervision of a senior investigator (VP). The study protocol was prospectively developed and submitted for registration in the International Prospective Register of Systematic Reviews (PROSPERO), where it is currently under review. The review process was carried out from September 2025 to April 2026, during which the final revisions were completed. A comprehensive and systematic literature search was conducted across the electronic databases PubMed, Scopus, and Google Scholar to identify relevant studies on toe walking in children with Autism Spectrum Disorder. The search strategy was developed *a priori* in accordance with PRISMA recommendations, combining controlled vocabulary (Medical Subject Headings, MeSH) and free-text terms related to the target population, condition, and interventions of interest. Boolean operators (“AND,” “OR”) were systematically applied to optimize both sensitivity and specificity of the search. The PubMed search string was defined as follows: (“Autism Spectrum Disorder”[MeSH] OR autism OR ASD) AND (“Toe Walking” OR toe-walking OR “equinus gait”) AND (treatment OR therapy OR rehabilitation OR surgery OR intervention), with filters applied for English language and a publication time frame limited to the last 20 years. A comparable strategy was adapted for Scopus using the TITLE-ABS-KEY field: (autism OR “autism spectrum disorder”) AND (“toe walking” OR “toe-walking” OR “equinus gait”) AND (treatment OR therapy OR rehabilitation OR surgery OR intervention), applying the same language and temporal restrictions. For Google Scholar, the following search string was employed: (“autism spectrum disorder” OR autism) AND (“toe walking” OR “toe-walking” OR “equinus gait”) AND (treatment OR therapy OR rehabilitation OR surgery). To ensure comprehensiveness and minimize the risk of missing relevant studies, the reference lists of all included articles were manually screened, and additional records were considered where appropriate. Articles published over the past 20 years related to the topic of interest were selected for inclusion in this review, ensuring a comprehensive analysis of both historical and contemporary approaches to the treatment of toe walking in children with Autism Spectrum Disorder. The search began in PubMed and subsequently extended to the other databases. Additionally, the reference lists of all eligible articles were manually reviewed to identify further relevant studies.

### Study selection

**Figure 1:**
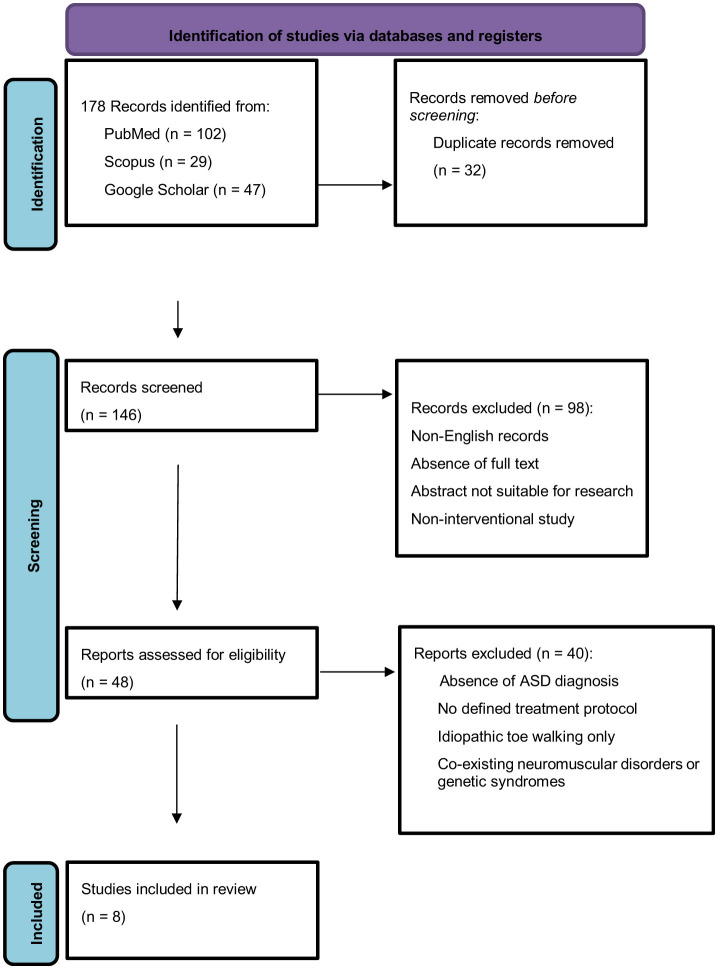
PRISMA flow-chart diagram illustrating the selection process of reports included in this review.

A total of 178 records were initially identified. After removal of 32 duplicates, 146 records were identified and screened through systematic searches in PubMed, Scopus, and Google Scholar. After removing duplicates and screening titles and abstracts, 98 records were excluded because they were not written in English; full text was unavailable; the abstract was not suitable for the research; the study did not report an intervention. Of the remaining 48 full-text articles assessed for eligibility, 40 were excluded because they did not include patients with ASD, did not report a defined treatment protocol, focused exclusively on idiopathic toe walking, or included patients with co-existing neuromuscular disorders or genetic syndromes. Ultimately, eight studies met the full inclusion criteria and were incorporated into the final analysis, as depicted in the PRISMA flow diagram (Figure 1). Eligible studies included randomized controlled trials (RCTs) and non-randomized studies of interventions (NRSIs), with continuous consideration of potential bias, which is inherently higher in mixed-method systematic reviews. Only studies published in English within the past 20 years were considered. The inclusion criteria were: studies written in English; clinical diagnosis of toe walking in patients with ASD; adherence to follow-up protocols; adherence to treatment regimens; clear treatment protocols addressing toe walking in ASD. The exclusion criteria included: non-English articles; absence of full text; abstract not suitable for the research; non-interventional study; studies focusing on idiopathic toe walking only; absence of treatment protocol for ASD patients; absence of ASD diagnosis; co-existing neuromuscular disorders or genetic syndromes.

### Data extraction

From each included study, the following data were extracted: number of patients, diagnosis, mean age, sex, type of treatment, mean follow-up duration, study design, authorship, year of publication, and the country of origin. Data were independently extracted from all included studies by four reviewers (AC, EP, GC, Ad, and CP). Each reviewer worked in parallel to ensure accuracy and minimize potential bias. Any discrepancies between reviewers were resolved through discussion and, when necessary, by consultation with a senior author (VP) to reach consensus. To ensure the validity and reliability of the data, predefined inclusion and exclusion criteria were strictly applied during the study selection and data extraction process. A standardized data extraction form was used to collect relevant information, including study characteristics, participant demographics, intervention details, and outcomes. Additionally, cross-checking procedures were performed to verify the consistency and accuracy of the extracted data, thereby enhancing the methodological rigor and reproducibility of the review.

### Outcome classification and hierarchy

Because the included studies used heterogeneous outcome measures, treatment response was not considered equivalent across studies. To improve methodological rigor, outcomes were classified according to a predefined hierarchy. Tier 1 outcomes included direct clinical gait correction, such as resolution of toe walking, restoration of heel-toe gait, or normalization of gait pattern. Tier 2 outcomes included quantitative behavioral or observational measures, such as reduction in toe walking frequency or toe walking episodes. Tier 3 outcomes included biomechanical or functional surrogate measures, such as improvement in ankle range of motion, Foot Posture Index, plantar pressure, or other kinematic parameters without direct assessment of toe walking resolution. Tier 4 outcomes included administrative or diagnostic persistence outcomes, such as persistence or absence of toe walking diagnosis in database studies. Because reported outcomes differed substantially across studies and outcome tiers, aggregated mean percentages were used only as descriptive summaries of study-specific reported improvement rates. They were not calculated from a common outcome definition and should not be interpreted as pooled estimates of a shared clinical endpoint or as measures of comparative treatment effectiveness. Accordingly, study-specific reported improvement rates were summarized descriptively only. Studies reporting surrogate or administrative outcomes were not considered directly comparable with studies reporting clinical gait normalization. Therefore, the term “success” was used cautiously and, where appropriate, replaced by “reported improvement” or “treatment response according to the study-specific outcome.”

### Descriptive synthesis

Given the substantial clinical and methodological heterogeneity across the included studies, no formal meta-analysis or confirmatory comparative effectiveness analysis was performed. Study-specific reported improvement rates were extracted at the study-arm level and summarized descriptively according to intervention type and outcome hierarchy. No inferential statistical comparison was used to support the main conclusions. For transparency, ancillary unweighted study-level Welch's *t*-test analyses were reported in the Supplementary material only. These analyses were not adjusted for sample size, study design, outcome definition, follow-up duration, or risk of bias, and were interpreted only as descriptive, hypothesis-generating summaries.

### Risk of bias and certainty of evidence assessments

To ensure methodological rigor and transparency, a structured approach to risk of bias assessment and evidence grading was implemented across all included studies. This process was designed to systematically evaluate the internal validity of individual studies and to determine the overall certainty of the evidence supporting the reported outcomes. Bias risk assessment was independently conducted by reviewers (EP, AC, GC, and Ad), with the senior reviewer (VP) holding greater adjudicative authority in cases of disagreement. Each study was evaluated in duplicate, and discrepancies were resolved through consensus to minimize subjectivity and enhance methodological rigor. The risk of bias in non-randomized studies was evaluated using the Risk Of Bias In Non-randomized Studies of Interventions (ROBINS-I) tool (27), while RCTs were assessed using the Cochrane Risk of Bias Tool (RoB 2.0) (28) examining domains such as the randomization process, deviations from intended interventions, missing outcome data, outcome measurement, and selection of the reported result. Any bias in individual domains could potentially lead to overestimation or underestimation of treatment effects. The quality of evidence was graded using the Grading of Recommendations, Assessment, Development, and Evaluation (GRADE) framework, which offers a structured approach to evaluating the strength and consistency of healthcare recommendations (29). The GRADE system considers domains such as risk of bias, consistency, precision, and directness, and classifies the evidence into levels of certainty to guide clinical decision-making.

## Results

Among the eight included studies, six were classified as conservative interventions (30–36) and two as surgical treatments (25, 36). Three studies used a unimodal approach (32, 36) while six used a multimodal approach (30, 31, 33–36). Study designs included one randomized controlled trial (RCT) (32) and seven non-randomized studies of interventions (NRSIs) (25, 30, 31, 33–36), with risk of bias assessed via RoB 2.0 and ROBINS-I, respectively. The NRSIs included in this review comprised heterogeneous designs, including one retrospective database cohort study, two prospective observational studies, one case report, one retrospective surgical case series, and two single-case experimental designs. Across all studies, a total of 424 participants with ASD were analyzed, ranging from 2 to 16 years, with mean age ranging from 6 to 9 years across studies. Males accounted for 69.7% of the sample and females for 30.3%, reflecting typical ASD demographics, although sex data were incompletely reported across studies due to lack of documentation. Follow-up durations varied from immediate post-treatment to 60 months, with longer durations observed primarily in surgical or multimodal conservative protocols. The main characteristics of the included studies are summarized in Table 1. Detailed data extraction revealed a wide range of treatment modalities. Outcome measures were heterogeneous across the included studies and included direct clinical assessment of toe walking resolution, observational gait analysis, biomechanical parameters, and indirect administrative outcomes. This variability in outcome definitions may have contributed to the heterogeneity observed in treatment effectiveness. Because included studies reported heterogeneous outcomes, a single uniform definition of success could not be applied across all studies. Therefore, treatment response was extracted according to the outcome definition used in each original study and subsequently classified according to the predefined outcome hierarchy. Direct clinical gait normalization and observed reduction in toe walking were considered higher-level outcomes, whereas range of motion (ROM), Foot Posture Index, plantar pressure, and administrative persistence of diagnosis were considered indirect or surrogate outcomes. Consequently, reported improvement rates were not pooled as equivalent measures of the same endpoint. Conservative approaches included serial casting (36), physical therapy (36), sensorimotor exercise (32), and multimodal interventions such as GaitSpot exercises (35), auditory feedback and reinforcement (31), the Cast and Go protocol with botulinum toxin (30), motor and behavioral exercises (33), casting and AFO (35). Surgical treatments included tendo-Achilles lengthening (25) and combined orthopedic management (36).

**Table 1:** Summary of included studies on toe walking interventions in ASD.

References	ASD patients (*n*)	Sex (M/F)	Mean age (years)	Treatment	Follow-up	Approach	Study design
Manfredi et al. (30)	22	12/10	~7–10	Cast and Go (Botox + casting + AFO + rehab)	Up to 12 months	Multimodal	Prospective observational study
Semino et al. (33)	4	4/0	~4–6	Motor + behavioral therapy	Short-term (~2 weeks)	Multimodal	Single-case experimental design
Wilder et al. (31)	3	3/0	~4–6	Auditory feedback + reinforcement	Short-term	Multimodal	Single-case experimental design
Marcus et al. (34)	3	3/0	~8–9	GaitSpot + behavioral training	Short-term	Multimodal	Small observational study
Barkocy et al. (35)	1	1/0	7	Serial casting + AFO	Short-term	Multimodal	Case report
Leyden et al. (36) (PT)	287	NR	Pediatric (< 18)	Physical therapy alone	Up to 2 years	Unimodal	Retrospective database cohort study
Leyden et al. (36) (casting)	36	NR	Pediatric (< 18)	Serial casting alone	Up to 2 years	Unimodal	Retrospective database cohort study
Leyden et al. (36) (PT + casting)	27	NR	Pediatric (< 18)	Physical therapy + casting	Up to 2 years	Multimodal	Retrospective database cohort study
Kandhasamy et al. (32)	20	NR	~3–5	Sensorimotor exercises	8 weeks	Unimodal	Small-sample RCT
Lyons et al. (25) (ASD subgroup)	5	NR	~2.5–16	Achilles tendon lengthening	Long-term (2–5 years)	Surgical	Retrospective surgical case series
Leyden et al. (36) (surgery)	16	NR	Pediatric (< 18)	Surgical correction	Up to 2 years	Surgical	Retrospective database cohort study

To improve the methodological interpretation of heterogeneous outcomes, included studies were classified according to a predefined outcome hierarchy. Tier 1 included studies reporting direct clinical gait correction, such as restoration of heel-toe gait or clinical resolution of toe walking; therefore, Manfredi et al. (30) and Lyons et al. (25) were assigned to this level. Tier 2 included studies reporting direct behavioral or observational reduction of toe walking episodes, or kinematic gait improvement; Semino et al., (33) Wilder et al., (31) Marcus et al., (34) and Barkocy et al. (35) were classified in this category because they directly assessed changes in toe walking behavior or gait pattern, although often in small samples or single-case designs. Tier 3 included studies reporting only surrogate biomechanical outcomes, such as ankle range of motion or Foot Posture Index; Kandhasamy et al. was assigned to this level because it did not directly measure toe walking resolution (32). Tier 4 included studies based on administrative or diagnostic persistence outcomes; all Leyden et al. (36) treatment arms were classified in this category because persistence of toe walking diagnosis does not necessarily correspond to direct clinical gait assessment. The outcome hierarchy applied to the included studies is reported in Table 2. Therefore, treatment responses across tiers were interpreted descriptively and were not considered equivalent measures of success.

**Table 2:** Outcome hierarchy of the included studies.

References	Reported outcome	Outcome category	Tier	Interpretation
Manfredi et al. (30)	Clinical resolution of toe walking and restoration of heel-toe gait	Direct clinical gait outcome	1	Directly measures clinical correction of toe walking
Lyons et al. (25) ASD subgroup	Clinical correction of equinus deformity and restoration of heel-toe gait	Direct clinical gait outcome	1	Direct surgical gait correction outcome
Semino et al. (33)	Reduction of toe walking frequency and improvement in ankle ROM	Behavioral/observational gait outcome with associated biomechanical improvement	2	Directly assesses reduction in toe walking behavior, although in a small single-case design
Wilder et al. (31)	Reduction in toe-walking behavior based on observational frequency measures	Behavioral/observational gait outcome	2	Measures toe walking episodes directly through behavioral observation
Marcus et al. (34)	Reduction in toe-walking episodes	Behavioral/observational gait outcome	2	Directly measures reduction in toe walking behavior, but without full clinical gait normalization
Barkocy et al. (35)	Kinematic gait improvement, ankle dorsiflexion, gait normalization	Kinematic/clinical gait improvement	2	Includes gait improvement and normalization, but based on a single case with kinematic assessment
Kandhasamy et al. (32)	Improvement in ankle ROM and Foot Posture Index	Biomechanical surrogate outcome	3	Reports surrogate biomechanical improvement
Leyden et al. (36) PT alone	Persistence or absence of toe walking diagnosis	Administrative/diagnostic outcome	4	Based on diagnostic persistence, not direct gait assessment
Leyden et al. (36) casting alone	Persistence or absence of toe walking diagnosis	Administrative/diagnostic outcome	4	Administrative outcome; not equivalent to clinical gait normalization
Leyden et al. (36) PT + casting	Persistence or absence of toe walking diagnosis	Administrative/diagnostic outcome	4	Administrative outcome despite multimodal conservative treatment
Leyden et al. (36) surgery	Persistence or absence of toe walking diagnosis	Administrative/diagnostic outcome	4	Surgical outcome assessed indirectly through diagnosis persistence

The following descriptive synthesis summarizes reported treatment responses for conservative and surgical interventions in children with ASD-related toe walking. A total of eight clinical studies were included, yielding nine conservative treatment groups and two surgical approaches. Importantly, the study by Leyden et al. (36) was stratified into distinct conservative treatment modalities, thereby contributing three additional groups to the six derived from the remaining studies.

**Figure 2:**
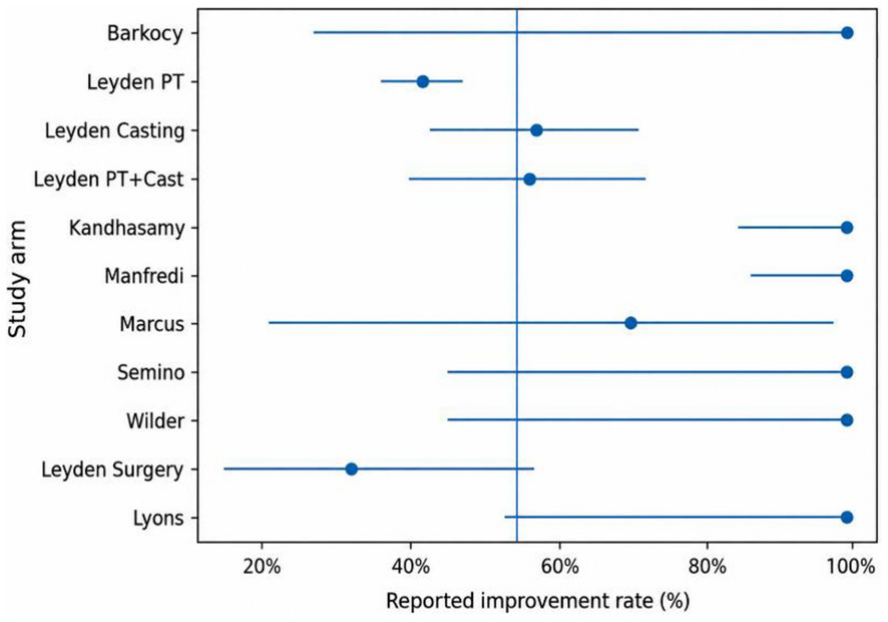
Descriptive plot of study-level reported improvement rates for conservative and surgical interventions. Values represent study-specific reported improvement rates based on heterogeneous outcome definitions. They should not be interpreted as pooled estimates or comparative effectiveness estimates.

Conservative treatment arms, modalities, study-specific reported improvement rates, and outcome 304 definitions are summarized in Table 3. Conservative interventions generally reported higher improvement rates, although outcome definitions and assessment methods were highly heterogeneous. Multimodal approaches generally showed numerically higher reported improvement rates in several small studies. The “Cast and Go” protocol described by Manfredi et al. (30) reported complete clinical resolution combining botulinum toxin, serial casting, orthoses, and multidisciplinary rehabilitation, with outcomes defined as clinical resolution of toe walking and restoration of a physiological heel-toe gait pattern. Similarly, Semino et al. (33) reported complete reduction of toe walking frequency using a structured multimodal motor-behavioral program assessing both reduction in toe walking frequency and improvements in ankle range of motion (ROM). Wilder et al. (31) also reported complete reduction in toe walking, although requiring reinforcement strategies in some patients; outcomes were based on direct observation of gait behavior and quantification of toe walking episodes using reinforcement-based paradigms. The case study by Barkocy et al. (35) reported clinically relevant kinematic improvement after combined casting and orthotic intervention, focusing on improvements in ankle dorsiflexion and normalization of gait pattern. In contrast, Marcus et al. (34) reported a more variable response, with an overall success rate of 66.7%, reflecting inter-individual differences in response to behavioral gait retraining and assessed through reduction in toe walking episodes. Within the study by Leyden et al., conservative treatments were stratified into three distinct arms, outcomes were based on the persistence of the toe walking diagnosis as an administrative outcome. This measure was classified as a Tier 4 outcome because it does not necessarily reflect direct clinical gait normalization and may be influenced by coding practices, follow-up patterns, and healthcare utilization. Physical therapy alone showed the lowest reported improvement rate of 36.2%, while serial casting alone achieved 52.8%, and the combined physical therapy plus casting group demonstrated a comparable improvement rate of 51.9% (36). These findings suggest variability in reported treatment response, but should be interpreted cautiously because the outcome was administrative rather than clinical. Among unimodal interventions, Kandhasamy et al. reported improvement in surrogate biomechanical outcomes, including ankle range of motion and Foot Posture Index (32). However, because direct toe walking resolution was not assessed, this study was not considered equivalent to studies reporting clinical gait normalization. Overall, conservative treatment arms showed a wide distribution of outcomes, ranging from 36.2 to 100%, with higher and more consistent improvement rates observed in multimodal interventions. Surgical treatment arms, study-specific reported improvement rates, and outcome definitions are summarized in Table 4. Surgical interventions showed heterogeneous results across studies. The ASD subgroup in Lyons et al. (25) reported complete clinical correction, with all patients achieving restoration of a physiological gait pattern following Achilles tendon lengthening. In contrast, the surgical cohort within Leyden et al. (36) demonstrated a lower reported improvement rate of 25%, based on persistence of toe walking diagnosis over time. These findings reflect substantial variability in surgical outcomes, likely influenced by patient selection, baseline severity, and the presence of neurodevelopmental factors associated with ASD. Reported treatment responses varied widely across both conservative and surgical treatment arms. The study-level reported improvement rates for conservative and surgical interventions are shown descriptively in Figure 2. Conservative treatment arms showed study-specific reported improvement rates ranging from 36.2 to 100%, whereas surgical treatment arms showed reported improvement rates ranging from 25.0 to 100%. However, these values were based on heterogeneous outcome definitions, including direct clinical gait correction, behavioral frequency reduction, biomechanical surrogate measures, and administrative persistence of diagnosis. Therefore, these findings were summarized descriptively and were not used to support inferential claims regarding comparative effectiveness.

**Table 3:** Summary of conservative treatments, modality, study-specific reported improvement rates, and outcome definitions.

References	*n* ASD	Treatment	Modality	Reported improvement rate (%)	Outcome
Barkocy et al. (35)	1	Serial casting + AFO	Multimodal	90	Kinematic gait improvement (ankle dorsiflexion, gait normalization)
Leyden et al. (36)—PT alone	287	Physical therapy	Unimodal	36.2	Persistence of toe walking diagnosis (administrative outcome)
Leyden et al. (36)—casting alone	36	Serial casting	Unimodal	52.8	Persistence of toe walking diagnosis (administrative outcome)
Leyden et al. (36)—PT + casting	27	Physical therapy + casting	Multimodal	51.9	Persistence of toe walking diagnosis (administrative outcome)
Kandhasamy et al. (32)	20	Sensorimotor exercises	Unimodal	100.0^*^	Ankle range of motion (ROM) and Foot Posture Index (indirect outcome)
Manfredi et al. (30)	22	Cast and Go (Botox + casting + AFO + rehab)	Multimodal	100.0	Clinical resolution of toe walking and restoration of heel-toe gait
Marcus et al. (34)	3	GaitSpot + behavioral training	Multimodal	66.7	Reduction in toe walking episodes (behavioral outcome)
Semino et al. (33)	4	Motor + behavioral treatment package	Multimodal	100.0	Reduction of toe walking frequency and improvement in ROM
Wilder et al. (31)	3	Auditory feedback + reinforcement	Multimodal	100.0	Reduction in toe walking behavior (observational outcome)

^*^Ramya = indirect outcome (ROM + FPI), not direct toe walking resolution.

Reported improvement rates are based on study-specific outcome definitions and are not directly comparable across studies. These values are descriptive only and do not represent pooled estimates of a common clinical endpoint.

**Table 4:** Summary of surgical treatments, study-specific reported improvement rates, and outcome definitions.

References	*n* ASD	Treatment	Reported improvement rate (%)	Outcome
Leyden et al. (36)—surgery	16	Orthopedic surgical correction	25.0	Persistence of toe walking diagnosis (administrative outcome)
Lyons et al. (25)—ASD subgroup	5	Tendo-Achilles lengthening (TAL)	100.0	Clinical correction of equinus deformity and restoration of heel-toe gait

Reported improvement rates are based on study-specific outcome definitions and are not directly comparable across studies. These values are descriptive only and do not represent pooled estimates of a common clinical endpoint.

**Figure 3:**
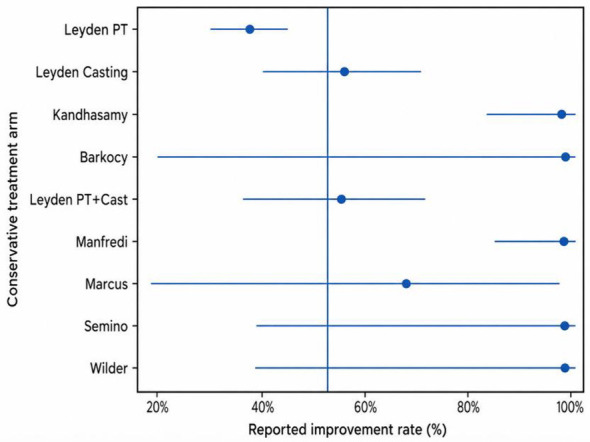
Descriptive plot of study-level reported improvement rates for unimodal and multimodal conservative interventions. Values represent study-specific reported improvement rates based on heterogeneous outcome definitions. They should not be interpreted as pooled estimates or comparative effectiveness estimates.

Reported treatment responses were then summarized descriptively according to intervention type, either unimodal or multimodal conservative interventions. The study-level reported improvement rates for unimodal and multimodal conservative interventions are shown descriptively in Figure 3. Only studies investigating conservative management were included in this comparison. Each study was classified according to the type of intervention as either unimodal, when a single therapeutic approach was applied (e.g., physical therapy, serial casting, or sensorimotor exercises), or multimodal, when two or more therapeutic components were combined (e.g., casting with orthotic management, motor training with behavioral reinforcement, or integrated rehabilitation protocols). Data were extracted at the study-arm level, and improvement rates were defined based on reported outcomes, including reduction or resolution of toe walking, improvement in gait pattern, or relevant functional or biomechanical parameters. Among conservative interventions, multimodal treatment arms generally reported numerically higher and more consistent study-specific improvement rates than unimodal approaches. Nevertheless, this observation should be interpreted cautiously because multimodal studies were heterogeneous in design, treatment components, follow-up duration, and outcome definitions. Moreover, the available evidence does not clarify whether the reported improvements resulted from synergistic effects between treatment components or from a single dominant intervention within the multimodal protocol. Therefore, the apparent pattern favoring multimodal strategies was considered descriptive and hypothesis-generating only.

Multimodal interventions, including combinations of casting, orthotic management, rehabilitation, and behavioral strategies, were associated with numerically higher and more consistent reported improvement rates in several studies. In contrast, unimodal approaches, such as physical therapy or isolated casting, exhibited greater variability in outcomes. For transparency, ancillary unweighted study-level Welch's *t*-test analyses are reported in Supplementary Table S1. These analyses were not used to support inferential conclusions and should be interpreted only as descriptive, hypothesis-generating summaries. The heterogeneity in study design, outcome definitions, and measurement methods, together with the use of aggregated data rather than individual patient data, limits the ability to draw definitive conclusions regarding the superiority of one treatment approach over another.

Domain-level risk-of-bias assessment showed that the main methodological limitations were related to confounding, absence of control groups, small sample sizes, indirect or non-standardized outcome measures, and lack of blinded outcome assessment. The single randomized controlled trial by Kandhasamy et al., assessed using the Cochrane RoB 2.0 tool, was judged as presenting some concerns, mainly related to limitations in outcome measurement and insufficient reporting of allocation concealment (32). The domain-level RoB 2 assessment for the randomized controlled trial is reported in Table 5. All remaining studies were non-randomized studies of interventions (NRSIs) and were evaluated using the ROBINS-I framework. Among these, the retrospective database study by Leyden et al. (36) was classified at serious risk of bias due to confounding and reliance on administrative outcome measures. The prospective case series by Manfredi et al. (30) and the retrospective case series by Lyons et al. (25) were also judged at serious risk of bias, primarily due to the absence of control groups and high susceptibility to outcome measurement bias. The remaining studies, including those by Semino et al., (33) Wilder et al., (31) Marcus et al., (34) and Barkocy et al., (35) were classified at critical risk of bias due to very small sample sizes, single-case or case report designs, lack of comparator groups, and high susceptibility to measurement bias. Overall, 12.5% of studies were judged as having some concerns, 37.5% as serious, and 50.0% as critical risk of bias. The domain-level ROBINS-I assessment for the non-randomized studies is reported in Table 6. The certainty of evidence, assessed using the GRADE framework for the main descriptive comparisons, was rated as very low. This result was mainly attributable to serious risk of bias, small sample sizes, heterogeneous study designs, indirect outcome measures, and inconsistent definitions of treatment response. Therefore, confidence in the observed estimates is limited, and the reported differences between treatment categories should be interpreted as exploratory and hypothesis-generating rather than as evidence of treatment superiority.

**Table 5:** Domain-level risk-of-bias assessment according to RoB 2 for the randomized controlled trial.

References	Randomization process	Deviations from intended interventions	Missing outcome data	Measurement of outcome	Selection of reported result	Overall risk of bias
Kandhasamy et al. (32)	Some concerns	Some concerns	Low	Some concerns	Some concerns	Some concerns

**Table 6:** Domain-level risk-of-bias assessment according to ROBINS-I for non-randomized studies.

References	Confounding	Selection of participants	Classification of interventions	Deviations from intended interventions	Missing data	Measurement of outcomes	Selection of reported result	Overall risk of bias
Manfredi et al. (30)	Serious	Moderate	Moderate	Moderate	Low	Serious	Moderate	Serious
Semino et al. (33)	Critical	Serious	Moderate	Serious	Low	Critical	Serious	Critical
Wilder et al. (31)	Critical	Serious	Moderate	Serious	Low	Critical	Serious	Critical
Marcus et al. (34)	Critical	Serious	Moderate	Serious	Low	Critical	Serious	Critical
Barkocy et al. (35)	Critical	Critical	Moderate	Serious	Low	Critical	Serious	Critical
Leyden et al. (36)	Serious	Moderate	Moderate	Moderate	Moderate	Serious	Moderate	Serious
Lyons et al. (25)	Serious	Serious	Moderate	Moderate	Moderate	Serious	Moderate	Serious

## Discussion

Toe walking is a gait pattern characterized by the absence of heel strike during ambulation and is observed in approximately 5%−12% of typically developing children under the age of five (37). While many cases resolve spontaneously, persistent toe walking beyond early childhood may indicate underlying neurological or developmental conditions. Among these, Autism Spectrum Disorder (ASD) is strongly associated, with studies reporting a prevalence of toe walking in up to 41%−68% of children with ASD (38, 39). Toe walking can be idiopathic, neuromuscular, or sensory-based in origin, with distinct etiological and therapeutic implications (39). Its persistence may lead to functional limitations, musculoskeletal complications, and social impairment. The treatment of toe walking varies depending on its underlying cause, ranging from observation and physiotherapy in idiopathic cases to orthotic management, casting, botulinum toxin injections, and surgery in persistent or neurodevelopmental forms. Aquatic-based interventions have been reported to improve muscular strength, endurance, motor skills, and flexibility in children with ASD. Although evidence regarding long-term effects remains limited, these findings support the hypothesis that the aquatic environment may be particularly beneficial for this population. The reduced sensory overload, combined with structured and predictable settings, may facilitate motor learning and promote social interaction in children with ASD (40). Aquatic exercise programs incorporating structured learning strategies have also been reported to improve physical abilities, social skills, and quality of life in children with ASD, with caregiver reports suggesting potential relational and behavioral benefits (41). Recent literature has suggested a potential role for multimodal conservative approaches, particularly when tailored to the sensory and motor profiles of children with ASD, before considering surgical correction (21). Surgical intervention, particularly Achilles tendon lengthening, has been shown to be effective in correcting persistent toe walking; however, it is generally recommended only in selected cases, such as severe deformities or failure of conservative treatment (18). The present study provides an exploratory synthesis of the available evidence on the management of toe walking in children with ASD, comparing conservative and surgical approaches as well as unimodal and multimodal treatment strategies. An important issue emerging from this review is whether ASD-associated toe walking should be considered simply a variant of idiopathic toe walking or rather a partially distinct clinical phenotype. Although both conditions may share a similar biomechanical presentation, toe walking in children with ASD may be influenced by additional neurodevelopmental mechanisms, including atypical sensory processing, altered proprioceptive integration, motor coordination difficulties, motor planning abnormalities, behavioral rigidity, and reinforcement-related gait patterns (42–45). Recent evidence suggests that toe walking in ASD may be associated with broader neurodevelopmental severity, including motor and language impairment and autism symptom severity, supporting the hypothesis that ASD-related toe walking may reflect a more complex clinical phenotype than idiopathic toe walking alone (42, 43). Therefore, treatment strategies focused exclusively on ankle dorsiflexion or correction of equinus may be insufficient if sensory, motor, and behavioral drivers remain unaddressed. A major limitation of the available literature is the lack of standardized outcome reporting. The included studies assessed treatment response using substantially different endpoints, ranging from direct clinical gait correction to behavioral frequency counts, biomechanical surrogate measures, and administrative persistence of diagnosis. These outcomes are not equivalent and should not be interpreted as measuring the same construct. For this reason, the present review introduced an outcome hierarchy and interpreted treatment responses according to the directness and clinical relevance of each endpoint. Studies reporting only ROM, Foot Posture Index, or administrative diagnosis persistence were considered less directly informative regarding true toe walking resolution than studies reporting clinical gait normalization or observed reduction in toe walking frequency. In the descriptive synthesis, conservative and surgical treatment arms showed heterogeneous reported improvement rates. These findings should not be interpreted as evidence that one approach is more effective than the other, because surgical candidates are likely to represent a subgroup with greater baseline severity, fixed equinus contractures, longer symptom duration, or failure of previous conservative management. Therefore, differences in reported outcomes may reflect patient selection, baseline severity, and outcome definition rather than true comparative effectiveness. In Leyden et al., (36) the surgical cohort showed a reported improvement rate of only 25% when assessed through persistence of diagnosis, an administrative outcome that may not directly reflect clinical gait correction. Conversely, the ASD subgroup in Lyons et al. (25) demonstrated a complete clinical resolution following Achilles tendon lengthening, suggesting that surgical correction can be effective in selected patients. However, the small sample size (*n* = 5) limits the strength of this conclusion. This finding may reflect selection bias, as surgical candidates are likely to represent more severe or refractory cases. Additionally, the use of administrative data as an outcome measure further complicates interpretation. Surgical management, particularly Achilles tendon lengthening, has been shown to provide effective correction in selected cases, especially in severe or refractory toe walking (46). However, this result should be interpreted cautiously, as such patients are likely to represent more severe or treatment-resistant cases. Conversely, the favorable results observed in the ASD subgroup of Lyons et al. (25) suggest that surgical intervention may be effective in selected patients, particularly when conservative management has failed. Overall, these findings support the notion that surgical treatment remains a valid option but should be carefully considered and possibly reserved for refractory cases. Previous studies have reported variable outcomes and recurrence rates, particularly in neurodevelopmental populations, highlighting the risk of recurrence and emphasizing the importance of patient selection (25). A large retrospective study reported that children with ASD have a higher prevalence of persistent toe walking than children without ASD, although the relationship between ASD diagnosis and subsequent surgical intervention may be influenced by confounding factors (47). Leyden et al. (36) reported that children with ASD and toe walking received surgical correction more frequently than typically developing children with toe walking, suggesting that treatment pathways may differ according to underlying neurodevelopmental status and clinical complexity. Consequently, differences in outcomes between conservative and surgical cohorts may reflect patient selection and baseline severity rather than true differences in treatment effectiveness.

A descriptive observation of this review is that multimodal conservative interventions generally reported higher and more consistent reported improvement rates than unimodal approaches. Some studies have reported favorable outcomes with multimodal interventions compared with single-modality approaches, particularly in neurodevelopmental conditions such as ASD. Toe walking in ASD is likely driven by a complex interaction of motor, sensory, and behavioral factors; therefore, integrated treatment strategies combining biomechanical correction, motor training, and behavioral reinforcement may theoretically address multiple contributing mechanisms (21, 48, 49). Furthermore, current studies do not clarify whether favorable outcomes are attributable to synergistic effects between treatment components or to a single dominant intervention within the multimodal protocol. The study by Manfredi et al. reported a complete resolution using the “Cast and Go” protocol, which combines botulinum toxin injections, serial casting, orthotic bracing, and multidisciplinary rehabilitation. In this study botulinum toxin was used as part of a multimodal protocol to reduce calf muscle overactivity and facilitate ankle dorsiflexion. This pharmacological modulation was intended to enhance the effectiveness of serial casting and orthotic management, thereby improving gait retraining and overall treatment outcomes (30). The strength of this study lies in its structured and comprehensive approach, integrating biomechanical correction with functional rehabilitation. However, its observational design and lack of a control group limit the generalizability of the findings. Similarly, Semino et al. (33) achieved a complete resolution using a multimodal intervention combining motor training and behavioral strategies. This study highlights the potential relevance of reinforcement-based techniques in modifying gait patterns. Nonetheless, its single-case experimental design and small sample size (*n* = 4) introduce a high risk of bias. The study by Wilder et al. (31) also demonstrated a complete reduction in toe walking, primarily through auditory feedback and reinforcement strategies. These findings emphasize the role of behavioral conditioning in ASD-related gait abnormalities. The necessity of continuous reinforcement and the absence of long-term follow-up raise concerns regarding durability of the effect. In contrast, Marcus et al. (34) reported a 66.7% success rate, reflecting a more variable response to behavioral interventions. While the study supports the effectiveness of auditory feedback systems (GaitSpot), it also highlights inter-individual variability and the potential need for adjunctive therapies. The case report by Barkocy et al. (35) demonstrated an optimal improvement with serial casting combined with orthotic management. Although clinically relevant, the single-patient design significantly limits external validity. Within the large database study by Leyden et al., (36) the multimodal subgroup (physical therapy combined with casting) achieved a 51.9% success rate, lower than other multimodal studies. This discrepancy may be explained using an indirect outcome measure based on persistence of diagnosis, as well as the inclusion of more severe or treatment-resistant cases. More broadly, multimodal exercise programs have been reported to influence loading rate and plantar pressure distribution during walking in boys with ASD. The work of Dehghani suggest that Sports, Play, and Active Recreation for Kids (SPARK) has the potential to alter the plantar pressure distribution during walking through a correction of toe walking in ASD children (50). Unimodal treatments demonstrated more variable outcomes, suggesting that single-modality approaches may be insufficient to address the complexity of toe walking in ASD. Previous literature supports the effectiveness of conservative interventions in managing toe walking, particularly in early stages (37). Studies on idiopathic toe walking have described the natural history and potential role of non-operative strategies such as casting and orthotic management, although extrapolation to ASD-related toe walking should be cautious (38). The randomized controlled trial by Kandhasamy et al. reported a success rate of approximately 100%, with significant improvements in foot posture and ankle range of motion following sensorimotor exercises (32). Despite its higher methodological quality, the study did not directly assess toe walking reduction, limiting its applicability to gait normalization outcomes. The unimodal arms of Leyden et al. further illustrate this variability. Physical therapy alone resulted in a low success rate of 36.2%, while serial casting alone achieved 52.8% (36). Multimodal treatments integrate multiple therapeutic domains, including biomechanical correction (casting, orthoses), motor training, sensory input, and behavioral reinforcement. This comprehensive approach appears particularly suited to ASD populations, in which toe walking is likely driven by a complex interplay of motor, sensory, and behavioral factors. The included studies illustrate the diversity of therapeutic approaches. Behavioral interventions, such as those described by Wilder et al. (31) and Marcus et al., (34) demonstrated that toe walking can be modulated through reinforcement-based strategies, highlighting the role of automatic and sensory reinforcement mechanisms. Similarly, multimodal rehabilitation protocols, such as the “Cast and Go” approach described by Manfredi et al., (30) achieved high success rates by combining pharmacological, mechanical, and rehabilitative components. Conversely, the randomized controlled trial by Kandhasamy et al. demonstrated improvements in ankle range of motion and foot posture but did not directly assess gait normalization (32). Therefore, while sensorimotor exercises appear beneficial, their effectiveness in eliminating toe walking remains uncertain. Taken together, the existing literature suggests that conservative and multimodal interventions may be reasonable initial options, particularly when fixed contractures are absent; however, this interpretation remains preliminary. Although multimodal conservative interventions appear promising, the mechanisms responsible for their reported benefits remain uncertain. Protocols combining serial casting, orthoses, botulinum toxin, rehabilitation, and behavioral strategies may theoretically address the multifactorial pathogenesis of ASD-related toe walking; however, current studies do not clarify whether improvement derives from a true synergistic effect between treatment components or from one dominant intervention within the protocol (30, 31, 33). For example, the Cast and Go protocol combines botulinum toxin, casting, orthoses, and rehabilitation, while behavioral protocols may combine motor exercises, reinforcement, feedback, and precision teaching (30, 33). Because most available studies lack factorial designs or comparator arms isolating each treatment component, the apparent advantage of multimodal strategies should be interpreted as a preliminary observation rather than evidence of additive or synergistic efficacy.

This study has several important limitations. The analysis was conducted at the study-arm level using aggregated data rather than individual patient data. Ancillary Welch's *t*-test analyses reported in the Supplementary material were limited by their unweighted study-level nature and were not used to support inferential conclusions regarding comparative effectiveness. Because the included studies used non-equivalent endpoints across different outcome tiers, all mean percentages reported in this review should be regarded only as descriptive summaries. They do not represent pooled estimates of a common clinical endpoint and should not be used to infer comparative treatment effectiveness. No robust inference can be made regarding the relative effectiveness of conservative vs. surgical treatment or unimodal vs. multimodal conservative strategies. Follow-up duration was limited in several included studies, particularly those evaluating behavioral or rehabilitation-based interventions. Short-term reductions in toe walking frequency may not reflect sustained gait correction, especially in children with sensory processing or neurodevelopmental comorbidities, in whom recurrence has been reported after treatment (24). Therefore, long-term maintenance of heel-toe gait, absence of recurrence, avoidance of retreatment, and prevention of fixed contractures should be considered more clinically meaningful outcomes than immediate post-treatment improvement. Outcome definitions were highly heterogeneous and included direct clinical gait correction, behavioral frequency reduction, biomechanical surrogate measures, and administrative persistence of diagnosis. These endpoints are not equivalent and cannot be assumed to reflect the same construct of treatment success. Reductions in toe walking frequency or improvements in ROM and Foot Posture Index may not necessarily translate into better functional mobility, quality of life, participation, pain, endurance, or prevention of long-term musculoskeletal complications. Finally, the limited number of surgical studies in ASD populations further restricts the strength of conclusions regarding surgical efficacy. Risk of bias and certainty of evidence were assessed through a two-step procedure based on study design. First, the only randomized controlled trial included in the review (1/8 studies, 12.5%) was evaluated with the Cochrane Risk of Bias 2.0 (RoB 2.0) tool, examining the randomization process, deviations from intended interventions, missing outcome data, outcome measurement, and selective reporting. This study was judged as presenting some concerns rather than low risk, because randomization was reported but allocation concealment and blinding procedures were insufficiently detailed, outcome assessment relied largely on indirect biomechanical measures rather than direct gait normalization, and no long-term follow-up was available. Second, the seven non-randomized studies of interventions (NRSIs; 87.5%) were assessed using the ROBINS-I framework across the domains of confounding, participant selection, intervention classification, deviations from intended interventions, missing data, outcome measurement, and selective reporting. Among these, the three retrospective cohort/database studies were judged at serious risk of bias, mainly due to confounding, absence of comparator balancing, and indirect or non-standardized outcome definitions, whereas the four case reports or single-case experimental studies were judged at critical risk of bias because of the absence of control groups, extremely small sample sizes, limited external validity, and likely measurement bias. Overall, this corresponds to 12.5% of studies with some concerns, 37.5% with serious risk of bias, and 50.0% with critical risk of bias. The certainty of evidence was then graded with the GRADE approach for the main comparative questions (conservative and surgical treatment; unimodal and multimodal conservative treatment). Because the body of evidence was dominated by NRSIs, showed substantial clinical and methodological heterogeneity, included several studies with indirect outcome measures, and remained markedly imprecise, the overall certainty of evidence was rated as very low. Therefore, although several studies reported high apparent improvement rates, confidence in the observed estimates remains very limited and the true effect is likely to differ substantially from the observed estimates. Overall, the available evidence suggests that conservative, surgical, unimodal, and multimodal strategies may all have a role in the management of ASD-related toe walking, depending on baseline severity, presence of fixed contracture, sensory-motor profile, and prior treatment history. Future prospective controlled studies should use standardized outcome definitions, stratify patients by baseline severity and contracture status, isolate treatment components when evaluating multimodal protocols, and include sufficiently long follow-up to assess recurrence and durability of gait correction. The comparative analyses performed in Supplementary material should be regarded as hypothesis-generating, and the findings should be interpreted with caution.

## Conclusion

In conclusion, both conservative and surgical interventions have been used for toe walking in children with ASD, but the current evidence does not allow definitive conclusions regarding comparative effectiveness. Multimodal conservative approaches appear promising and may be suitable for addressing the multifactorial nature of ASD-related toe walking. However, because the available evidence is heterogeneous and of very low certainty, no definitive conclusion can be drawn regarding the superiority of multimodal over unimodal strategies, or conservative over surgical treatment. The descriptive synthesis should therefore be considered hypothesis-generating only. Future high-quality prospective controlled studies using standardized clinical outcomes, adequate sample sizes, and long-term follow-up are required.

## Data Availability

The original contributions presented in the study are included in the article/Supplementary material, further inquiries can be directed to the corresponding author.

## References

[1] Posar A Visconti P. Autism Spectrum Disorder and the diagnostic and statistical manual of mental disorders—fifth edition (DSM-5): the experience of 10 years. Turk Arch Pediatr. (2023) 58:658–9. doi: 10.5152/TurkArchPediatr.2023.23149 37737228 PMC10724720

[2] Persico AM Ricciardello A Lamberti M Turriziani L Cucinotta F Brogna C . The pediatric psychopharmacology of Autism Spectrum Disorder: a systematic review - part I: the past and the present. Prog Neuropsychopharmacol Biol Psychiatry (2021) 110:110326. doi: 10.1016/j.pnpbp.2021.110326 33857522

[3] Gowen E Hamilton A. Motor abilities in autism: a review using a computational context. J Autism Dev Disord. (2013) 43:323–44. doi: 10.1007/s10803-012-1574-0 22723127

[4] Fournier KA Kimberg CI Radonovich KJ Tillman MD Chow JW Lewis MH . Decreased static and dynamic postural control in children with Autism Spectrum Disorders. Gait Posture (2010) 32:6–9. doi: 10.1016/j.gaitpost.2010.02.007 20400311 PMC2919314

[5] Papadopoulos D. Mothers' experiences and challenges raising a child with Autism Spectrum Disorder: a qualitative study. Brain Sci. (2021) 11:309. doi: 10.3390/brainsci11030309 33801233 PMC8001702

[6] Li Y Liu T Venuti CE. Development of postural stability in children with Autism Spectrum Disorder: a cross-sectional study. Int Biomech. (2021) 8:54–62. doi: 10.1080/23335432.2021.1968316 34414860 PMC8381937

[7] Provost B Heimerl S Lopez BR. Levels of gross and fine motor development in young children with Autism Spectrum Disorder. Phys Occup Ther Pediatr. (2007) 27:21–36. doi: 10.1080/J006v27n03_03 17613454

[8] Kilroy E Ring P Hossain A Nalbach A Butera C Harrison L . Motor performance, praxis, and social skills in Autism Spectrum Disorder and developmental coordination disorder. Autism Res. (2022) 15:1649–64. doi: 10.1002/aur.2774 35785418 PMC9543450

[9] Marco EJ Hinkley LBN Hill SS Nagarajan SS. Sensory processing in autism: a review of neurophysiologic findings. Pediatr Res. (2011) 69:48R−54R. doi: 10.1203/PDR.0b013e3182130c54 21289533 PMC3086654

[10] Dziuk MA Gidley Larson JC Apostu A Mahone EM Denckla MB Mostofsky SH. Dyspraxia in autism: association with motor, social, and communicative deficits. Dev Med Child Neurol. (2007) 49:734–9. doi: 10.1111/j.1469-8749.2007.00734.x 17880641

[11] Rinehart NJ Tonge BJ Bradshaw JL Iansek R Enticott PG Johnson KA. Movement-related potentials in high-functioning autism and Asperger's disorder. Dev Med Child Neurol. (2006) 48:272–7. doi: 10.1017/S0012162206000594 16542514

[12] Shetreat-Klein M Shinnar S Rapin I. Abnormalities of joint mobility and gait in children with Autism Spectrum Disorders. Brain Dev. (2014) 36:91–6. doi: 10.1016/j.braindev.2012.02.005 22401670

[13] Williams JHG Whiten A Singh T. A systematic review of action imitation in autistic spectrum disorder. J Autism Dev Disord. (2004) 34:285–99. doi: 10.1023/B:JADD.0000029551.56735.3a 15264497

[14] Sala R Amet L Blagojevic-Stokic N Shattock P Whiteley P. Bridging the gap between physical health and Autism Spectrum Disorder. Neuropsychiatr Dis Treat. (2020) 16:1605–18. doi: 10.2147/NDT.S251394 32636630 PMC7335278

[15] Shulman LH Sala DA Chu ML McCaul PR Sandler BJ. Developmental implications of idiopathic toe walking. J Pediatr. (1997) 130:541–6. doi: 10.1016/S0022-3476(97)70236-1 9108850

[16] Bremer E Crozier M Lloyd M. A systematic review of the behavioural outcomes following exercise interventions for children and youth with Autism Spectrum Disorder. Autism (2016) 20:899–915. doi: 10.1177/1362361315616002 26823546

[17] Case-Smith J. Systematic review of interventions to promote social-emotional development in young children with or at risk for disability. Am J Occup Ther. (2013) 67:395–404. doi: 10.5014/ajot.2013.004713 23791314

[18] Eastwood DM Menelaus MB Dickens DR Broughton NS Cole WG. Idiopathic toe-walking: does treatment alter the natural history? J Pediatr Orthop B. (2000) 9:47–9. doi: 10.1097/01202412-200001000-00010 10647110

[19] Sätilä H Beilmann A Olsén P Helander H Eskelinen M Huhtala H. Does botulinum toxin A treatment enhance the walking pattern in idiopathic toe-walking? Neuropediatrics (2016) 47:162–8. doi: 10.1055/s-0036-1582138 27089542

[20] Herrin K Geil M. A comparison of orthoses in the treatment of idiopathic toe walking: a randomized controlled trial. Prosthet Orthot Int. (2016) 40:262–9. doi: 10.1177/0309364614564023 25628380

[21] Schaaf RC Benevides T Mailloux Z Faller P Hunt J van Hooydonk E . An intervention for sensory difficulties in children with autism: a randomized trial. J Autism Dev Disord. (2014) 44:1493–506. doi: 10.1007/s10803-013-1983-8 24214165 PMC4057638

[22] Mortimer R Privopoulos M Kumar S. The effectiveness of hydrotherapy in the treatment of social and behavioral aspects of children with Autism Spectrum Disorders: a systematic review. J Multidiscip Healthc. (2014) 7:93–104. doi: 10.2147/JMDH.S55345 24520196 PMC3917923

[23] Fox A Deakin S Pettigrew G Paton R. Serial casting in the treatment of idiopathic toe-walkers and review of the literature. Acta Orthop Belg. (2006) 72:722–30. 17260610

[24] Zide JR Riccio AI Zak T Minopoli A Polk JL Shivers C. Recurrent toe walking in pediatric orthopedic patients: idiopathic vs concomitant sensory processing disorders. Foot Ankle Orthop. (2023) 8:2473011423S00189. doi: 10.1177/2473011423S00189 42244456

[25] Lyons D Ferderber J Fanelli M Hennrikus W. Outcomes following surgical correction of toe walking. Arch Orthop Rheumatol. (2020) 3:1–6. doi: 10.22259/2639-3654.0301001

[26] Page MJ McKenzie JE Bossuyt PM Boutron I Hoffmann TC Mulrow CD . The PRISMA 2020 statement: an updated guideline for reporting systematic reviews. BMJ (2021) 372:n71. doi: 10.1136/bmj.n71 33782057 PMC8005924

[27] Sterne JA Hernán MA Reeves BC Savović J Berkman ND Viswanathan M . ROBINS-I: a tool for assessing risk of bias in non-randomised studies of interventions. BMJ (2016) 355:i4919. doi: 10.1136/bmj.i4919 27733354 PMC5062054

[28] Nejadghaderi SA Balibegloo M Rezaei N. The Cochrane risk of bias assessment tool 2 (RoB 2) versus the original RoB: a perspective on the pros and cons. Health Sci Rep. (2024) 7:e2165. doi: 10.1002/hsr2.2165 38835932 PMC11147813

[29] Xie CX Machado GC. Clinimetrics: grading of recommendations, assessment, development and evaluation (GRADE). J Physiother. (2021) 67:66. doi: 10.1016/j.jphys.2020.07.003 32859566

[30] Manfredi F Riefoli F Coviello M Dibello D. The management of toe walking in children with Autism Spectrum Disorder: “cast and go.” *Children* (2022) 9:1477. doi: 10.3390/children9101477 36291413 PMC9600566

[31] Wilder DA Ertel H Hodges AC Thomas R Luong N. The use of auditory feedback and edible reinforcement to decrease toe walking among children with autism. J Appl Behav Anal. (2020) 53:554–62. doi: 10.1002/jaba.607 31292961

[32] Kandhasamy R Hemavathy S Jeyanthi D. Effect of sensorimotor exercises in reducing toe walking in Autistic Spectrum Disorder. Int J Res Rev. (2023) 10:315–9. doi: 10.52403/ijrr.20230743

[33] Semino M Riccio E Giannatiempo S Cavallini F Vascelli L. Evaluating a treatment package to reduce toe walking and improve ankle mobility in children with Autism Spectrum Disorder: a multi-component intervention. Behav Anal Pract. (2025) 18:206–20. doi: 10.1007/s40617-024-01035-8 40092327 PMC11904055

[34] Marcus A Sinnott B Bradley S Grey I. Treatment of idiopathic toe-walking in children with autism using GaitSpot auditory speakers and simplified habit reversal. Res Autism Spectr Disord. (2010) 4:260–7. doi: 10.1016/j.rasd.2009.09.012

[35] Barkocy M Dexter J Petranovich C. Kinematic gait changes following serial casting and bracing to treat toe walking in a child with autism. Pediatr Phys Ther. (2017) 29:270–4. doi: 10.1097/PEP.0000000000000404 28654502

[36] Leyden J Fung L Frick S. Autism and toe-walking: are they related? Trends and treatment patterns between 2005 and 2016. J Child Orthop. (2019) 13:340–5. doi: 10.1302/1863-2548.13.180160 31489038 PMC6701446

[37] Engström P Tedroff K. The prevalence and course of idiopathic toe-walking in 5-year-old children. Pediatrics (2012) 130:279–84. doi: 10.1542/peds.2012-0225d 22826572

[38] Kindregan D Gallagher L Gormley J. Gait deviations in children with Autism Spectrum Disorders: a review. Autism Res Treat. (2015) 2015:741480. doi: 10.1155/2015/741480 25922766 PMC4398922

[39] Ruzbarsky JJ Scher D Dodwell E. Toe walking: causes, epidemiology, assessment, and treatment. Curr Opin Pediatr. (2016) 28:40–6. doi: 10.1097/MOP.0000000000000302 26709689

[40] Pan CY Tsai CL Hsieh KW. Physical activity correlates for children with Autism Spectrum Disorders in middle school physical education. Res Q Exerc Sport (2011) 82:491–8. doi: 10.1080/02701367.2011.10599782 21957708

[41] Güeita-Rodríguez J Ogonowska-Slodownik A Morgulec-Adamowicz N Martín-Prades ML Cuenca-Zaldívar JN Palacios-Ceña D. Effects of aquatic therapy for children with Autism Spectrum Disorder on social competence and quality of life: a mixed methods study. Int J Environ Res Public Health (2021) 18:3126. doi: 10.3390/ijerph18063126 33803581 PMC8002945

[42] Camia M Sacco R Boncoddo M Bellomo F Cucinotta F Ricciardello A . Toe walking in children and adolescents with Autism Spectrum Disorder: relationship with sensory and motor functions, language, cognition, and autism severity. Res Autism Spectr Disord. (2024) 117:102457. doi: 10.1016/j.rasd.2024.102457

[43] Costanza C Gallai B Sorrentino M Gnazzo M Pisanò G Parisi L . The prevalence and clinical significance of toe walking in Autism Spectrum Disorder: a cross-sectional study in an Italian pediatric sample. Medicina (2025) 61:1346. doi: 10.3390/medicina61081346 40870390 PMC12387827

[44] Williams CM Tinley P Curtin M. Idiopathic toe walking and sensory processing dysfunction. J Foot Ankle Res. (2010) 3:16. doi: 10.1186/1757-1146-3-16 20712877 PMC2933674

[45] de Angeli LRA Serafim BLC Masquijo JJ. The autistic toe walking: a narrative review for interventions and comparison with idiopathic toe walking. Children (2025) 12:1198. doi: 10.3390/children12091198 41007062 PMC12468364

[46] Stricker SJ Angulo JC. Idiopathic toe walking: a comparison of treatment methods. J Pediatr Orthop. (1998) 18:289. doi: 10.1097/01241398-199805000-00003 9600550

[47] Chapek M Kessler J. The prevalence of persistent toe walking in children with and without Autism Spectrum Disorder and the odds of subsequent surgery. J Foot Ankle Surg. (2025) 64:16–20. doi: 10.1053/j.jfas.2024.08.005 39147358

[48] Sowa M Meulenbroek R. Effects of physical exercise on Autism Spectrum Disorders: a meta-analysis. Res Autism Spectr Disord. (2012) 6:46–57. doi: 10.1016/j.rasd.2011.09.001

[49] Baranek GT. Efficacy of sensory and motor interventions for children with autism. J Autism Dev Disord. (2002) 32:397–422. doi: 10.1023/A:1020541906063 12463517

[50] Dehghani M Jafarnezhadgero AA Darvishani MA Aali S Granacher U. Effects of an 8-week multimodal exercise program on ground reaction forces and plantar pressure during walking in boys with Autism Spectrum Disorder. Trials (2023) 24:170. doi: 10.1186/s13063-023-07158-7 36890589 PMC9993582

