# Supplementary material for: Comparative effectiveness of conservative and surgical interventions for toe walking in children with Autism Spectrum Disorder: a systematic review
Source: Front Med (Lausanne). 2026 Jul 20;13:1832930. doi: 10.3389/fmed.2026.1832930 (PMC13430461; doi:10.3389/fmed.2026.1832930)
Supplement: Supplementary file 1 [file Table_1.pdf]

Supplementary Material

Comparative Effectiveness of Conservative and Surgical Interventions for Toe Walking in Children with Autism Spectrum Disorder: A Systematic Review

Ancillary unweighted study-level Welch's t-test analyses were performed only as descriptive summaries of numerical differences between treatment categories. These analyses were not used to support inferential conclusions in the main manuscript.

Comparison	Group 1: mean reported improvement rate (%)	Group 1 SD	Group 2: mean reported improvement rate (%)	Group 2 SD	t value	p value	Interpretation
Conservative vs surgical	78.64	25.85	62.50	53.03	0.418	0.742	Ancillary unweighted study-level analysis; not inferential
Unimodal conservative vs multimodal conservative	63.00	33.10	86.43	21.53	-1.114	0.349	Ancillary unweighted study-level analysis; not inferential

Supplementary Table S1. Ancillary exploratory comparison of unweighted study-level reported improvement rates using Welch's t-test.

Values are based on unweighted study-level reported improvement rates. These analyses were not adjusted for sample size, study design, outcome definition, follow-up duration, or risk of bias. They should not be interpreted as evidence of comparative effectiveness, superiority, equivalence, or absence of effect.
